# The evolution of the coding exome of the *Arabidopsis* species - the influences of DNA methylation, relative exon position, and exon length

**DOI:** 10.1186/1471-2148-14-145

**Published:** 2014-06-25

**Authors:** Feng-Chi Chen, Trees-Juen Chuang, Hsuan-Yu Lin, Min-Kung Hsu

**Affiliations:** 1 Institute of Population Health Sciences, National Health Research Institutes, Miaoli County, Taiwan; 2 Department of Biological Science and Technology, National Chiao-Tung University, Hsinchu, Taiwan; 3 Department of Dentistry, China Medical University, Taichung, Taiwan; 4 Genomics Research Center, Academia Sinica, Taipei, Taiwan

**Keywords:** DNA methylation, Exon evolution, Evolutionary rate, Relative exon position, Exon length

## Abstract

**Background:**

The evolution of the coding exome is a major driving force of functional divergence both between species and between protein isoforms. Exons at different positions in the transcript or in different transcript isoforms may (1) mutate at different rates due to variations in DNA methylation level; and (2) serve distinct biological roles, and thus be differentially targeted by natural selection. Furthermore, intrinsic exonic features, such as exon length, may also affect the evolution of individual exons. Importantly, the evolutionary effects of these intrinsic/extrinsic features may differ significantly between animals and plants. Such inter-lineage differences, however, have not been systematically examined.

**Results:**

Here we examine how DNA methylation at CpG dinucleotides (CpG methylation), in the context of intrinsic exonic features (exon length and relative exon position in the transcript), influences the evolution of coding exons of *Arabidopsis thaliana*. We observed fairly different evolutionary patterns in *A. thaliana* as compared with those reported for animals. Firstly, the mutagenic effect of CpG methylation is the strongest for internal exons and the weakest for first exons despite the stringent selective constraints on the former group. Secondly, the mutagenic effect of CpG methylation increases significantly with length in first exons but not in the other two exon groups. Thirdly, CpG methylation level is correlated with evolutionary rates *(d*_S_, *d*_N_, and the *d*_N_/*d*_S_ ratio) with markedly different patterns among the three exon groups. The correlations are generally positive, negative, and mixed for first, last, and internal exons, respectively. Fourthly, exon length is a CpG methylation-independent indicator of evolutionary rates, particularly for *d*_N_ and the *d*_N_/*d*_S_ ratio in last and internal exons. Finally, the evolutionary patterns of coding exons with regard to CpG methylation differ significantly between *Arabidopsis* species and mammals.

**Conclusions:**

Our results suggest that intrinsic features, including relative exonic position in the transcript and exon length, play an important role in the evolution of *A. thaliana* coding exons. Furthermore, CpG methylation is correlated with exonic evolutionary rates differentially between *A. thaliana* and animals, and may have served different biological roles in the two lineages.

## Background

The evolution of the coding exome is a major driving force of functional divergence. In the past, a coding gene was considered as a basic unit for biological regulations and molecular functions. As such, in the majority of evolutionary studies, the “functional unit” targeted by natural selection is presumed to be a gene. However, with the advances in molecular biology and high-throughput sequencing technologies, it has gradually become clear that alternative transcript isoforms of the same gene (and the corresponding protein products) can be spatio-temporally regulated, and convey fairly divergent biological functions [1-5]. In other words, in many cases, a “transcript” rather than a “gene” is the biologically functional unit. The importance of transcript isoforms is particularly significant in complex organisms because they have highly developed networks of transcript/protein isoforms [5].

Transcript isoforms of the same gene differ from each other by alternatively spliced exonic regions. In cases where transcript isoforms convey distinct biological functions, the alternatively spliced exonic regions are crucial for the between-isoform functional divergences. These exonic regions should be accordingly targeted by natural selection. Therefore, the biological functions of alternative (and non-alternative) exonic sequences and the selection pressure thereon can be revealed by examining the evolutionary patterns of these sequences [6-11].

We previously examined the determinants of exonic evolutionary rates in mammals and *Arabidopsis* species. The biological factors that affect exonic evolutionary rates were found to differ between these two lineages [6,7]. In addition, we discovered that in mammals, the position of an exon (first, last, or internal exon) in the transcript is significantly associated with the evolution of the exonic sequence in accordance with the level of DNA methylation at CpG dinucleotides (“CpG methylation” in short) [12]. This is probably because the position of an exon is related to its biological function (or lack of function), thus making the exon selectively constrained for the function mediated by CpG methylation, or prone to the mutagenesis effect of CpG methylation [12]. However, whether this proposition is also true for *Arabidopsis* remains unexplored.

Plant coding exons differ from their mammalian counterparts in several aspects. Firstly, alternative RNA splicing is less well developed, and plays a less important role in exon evolution in plants than in mammals [5,7]. Secondly, on average, a plant gene includes fewer but longer exons than a mammalian gene [13-15]. Thirdly, the effective population sizes of plants (*Arabidopsis thaliana* as an example) are considerably larger than those of mammals (*e.g.* human and mouse) [16], giving rise to a higher efficiency of natural selection on plant exonic sequences. Given these differences, we expect the evolutionary patterns of *Arabidopsis* exons at different positions to diverge from those of their mammalian counterparts.

In this study, we systematically examined the mutational effects and of CpG methylation and its correlations with exonic evolutionary rates for *A. thaliana* coding exons at different positions. Our results indicate that first, last, and internal coding exons of *A. thaliana* have fairly different evolutionary patterns in this regard. The three exon groups diverge significantly in their liability to CpG methylation-related mutagenesis. Furthermore, the CpG methylation-evolutionary rate correlations differ significantly among the three exon groups. These correlations also differ significantly between *Arabidopsis* species and mammals. In addition, we found exon length to be a CpG methylation-independent indicator of exonic evolutionary rates in *Arabidopsis* species. Our results suggest that intrinsic exonic features (relative position and length) may be important determinants for the evolution of *A. thaliana* coding exons, and that CpG methylation may play different biological roles in the coding exons of mammals and *Arabidopsis* species.

## Results

### The mutagenic effect of CpG methylation for exons at different positions

**Figure 1:**
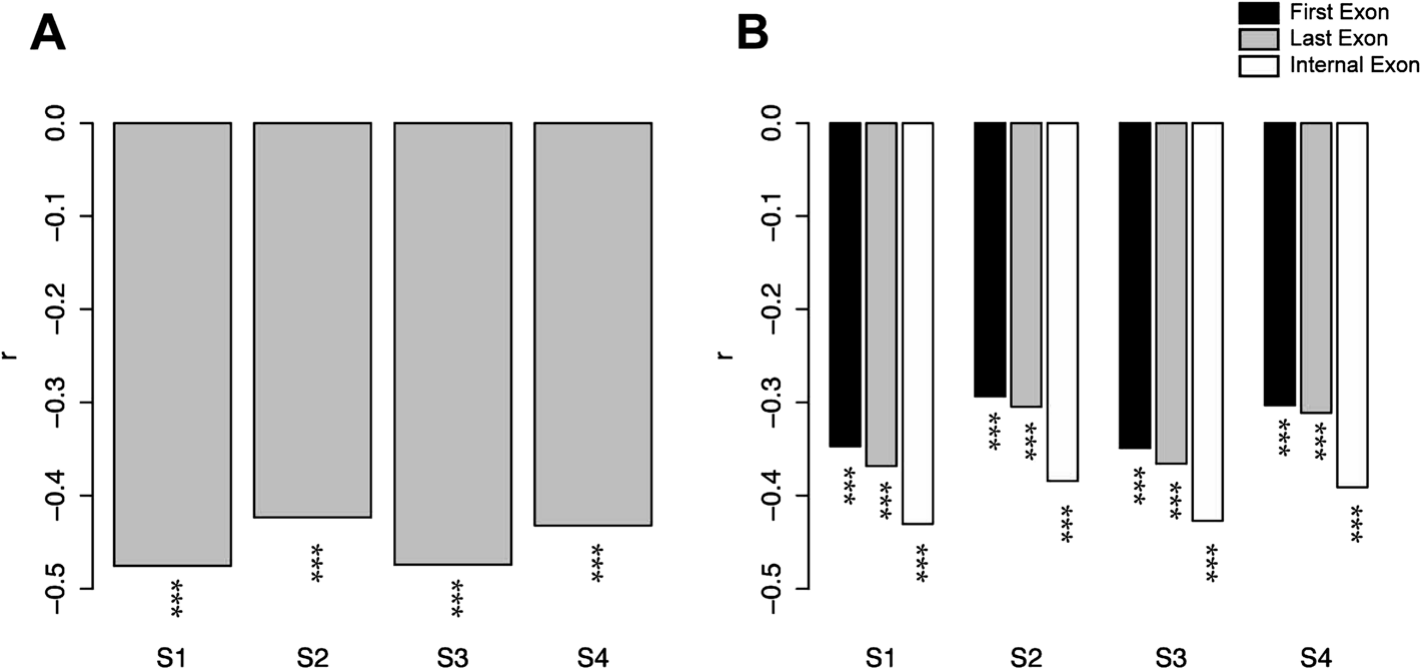
**Pearson’s coefficients of correlation between mCG density and the CpG O/E ratio in (A) different methylome datasets (S1 ~ S4); (B) first, last, and internal coding exons in different methylome datasets.** ***: *p* < 0.001.

To examine the mutagenic effect of CpG methylation on *A. thaliana* coding exons, we calculated the Pearson’s coefficient of correlation between the level of CpG methylation (represented by “mCG density”; see Methods) and the CpG O/E ratio (observed-to-expected ratio of the number of CpG dinucleotides, see Methods). Of note, here we do not include methylation at CHG or CHH sites (where “H” indicates A, C, or T) because these two types of methylation account for a minority of the plant methylome [17], and may have minor effects on the evolution of *Arabidopsis* exons. CpG methylation can significantly increase the rate of cytosine-to-thymine (C-to-T) transitions, leading to a decreased number of CpG dinucleotides. Therefore, mCG density is expected to be negatively correlated with the CpG O/E ratio. Furthermore, a larger absolute value of the coefficient of correlation (*r*) indicates a stronger mutagenic effect of DNA methylation [12]. Here the Pearson’s (rather than the Spearman’s) correlation is employed to show this quantitative relationship. Of note, when dealing with the methylome data, we applied a set of filtering criteria to ensure data quality and to reduce variations in the estimation of mCG density (Methods). These filtering criteria lead to differences in the number of analyzable exons among the four methylome datasets (S1 ~ S4 in Table 1). Interestingly, as shown in Figure 1A, despite the variations in the number of analyzable exons and the level of CpG methylation, mCG density is consistently negatively correlated with the CpG O/E ratio across datasets. And the *r* value falls within a relatively narrow range (−0.5 ~ −0.4). This observation confirms the mutagenic effect of CpG methylation on *A. thaliana* coding exons.

**Table 1:** The methylome datasets and the background exome dataset analyzed in this study

**Arabidopsis**	**Symbol**	**#Gene**	**#Exon**	**Bisulfite Seq. read dept**	**Average mCG density (per 100 Sampled CpG)**	**Average mCG density (per 100 Sampled bp)**	**#First/Last/Internal**	**Average length**
Col_wt	S1	10152	12649	14	17.3	4.8	6132/3182/3335	765.4
Col_wt	S2	14409	21230	97	16.3	4.7	9243/5316/6671	615.8
Col_wt	S3	9666	11758	17	17.1	4.8	5848/2944/2966	783.5
Col_wt	S4	14002	20199	65	16.7	4.7	6132/3182/3335	630.0
Col_O	Background	19500	79730	NA	NA	3.2	13933/12570/53227	282.9 ± 325.0

Note that the background dataset was not filtered for methylation data, but was filtered for the definitions of first, last, and internal exons and for the length threshold for calculations of evolutionary rates (see Methods). NA: not applicable. Note that the “Bisulfite Seq. read depth” is defined as the total length of the bisulfite sequencing reads divided by the size of the *A. thaliana* genome.

The next question to ask is whether the mutagenic effect of CpG methylation differs for first, last, and internal exons. To address this issue, we evaluated the Pearson’s correlations as described above separately for each of the three exon groups. Unexpectedly, as shown in Figure 1B, although the mCG-CpG O/E correlations remain negative across the three exon groups, the strongest and the weakest mutagenic effect occur, respectively, in internal and first exons. This is to the contrary of what was previously observed for mammals, where the strongest mutagenic effect of CpG methylation occurs in first exons, while the weakest in internal exons [12].

**Figure 2:**
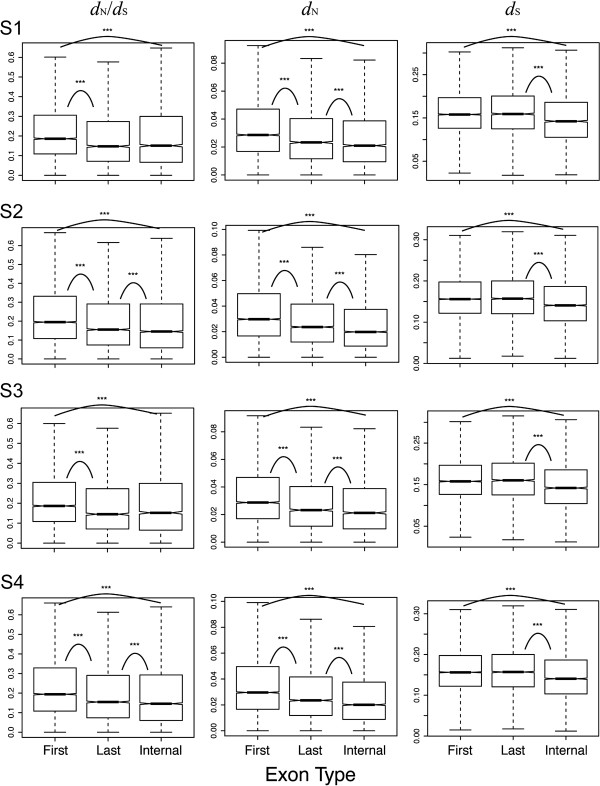
**The evolutionary rates (*d***_**N**_**/*d***_**S **_**ratio, *d***_**N**_**, and *d***_**S**_**) of first, last, and internal coding exons in different methylome datasets.** The curves with stars indicate statistically significant difference. ***: *p* < 0.001, by Wilcoxon Rank Sum Test.

We then ask how the variation in mCG-related mutagenic effect may have affected the evolutionary rates of the three exon groups. To this end, we first examined the evolutionary rates (*d*_N_, *d*_S_, and the *d*_N_/*d*_S_ ratio) separately for the three exon groups between *A. thaliana* and *A. lyrata* based on datasets S1 ~ S4 (Table 1). The four sperm methylome datasets used here are appropriate for this evolutionary analysis because only the mutations that occur in germ line cells can be propagated over generations, thus leaving observable changes in the inter-species comparison. Figure 2 shows that among the three exon groups, first exons have the largest median *d*_N_ and *d*_N_/*d*_S_ ratio, followed by last exons, and finally by internal exons (although the last-internal differences in *d*_N_/*d*_S_ are statistically insignificant in S1 and S3). For *d*_S_, internal exons have the lowest median value, and first and last exons have similar values.

The evolutionary rate profiles apparently are inconsistent with the mCG-related mutagenic effect profiles in first, last, and internal exons. Specifically, in internal exons, we observe the co-occurrence of a high median mCG density (Additional file 1), a strong mutagenic effect of CpG methylation (Figure 1B), and low evolutionary rates as compared with the other two exon groups (Figure 2). One possible explanation is that the strong selection pressure imposed on internal exons has significantly constrained the mCG-related mutations from occurring in this exon group. This appears to be true judging from the low *d*_N_/*d*_S_ ratio in internal exons as compared with the other two exon groups (Figure 2). Interestingly, the median *d*_S_ is also the lowest in internal exons, suggesting that synonymous substitutions are subject to strong purifying selection in this exon group.

### The correlations between CpG methylation and exonic evolutionary rates

**Figure 3:**
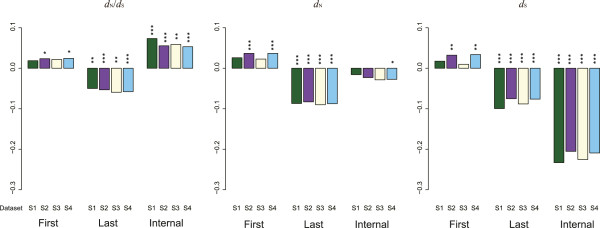
**The Spearman’s coefficients of correlation between mCG density and the *d***_**N**_**/*d***_**S **_**ratio, *d***_**N**_**, and *d***_**S **_**based on different methylome datasets.** *: *p* < 0.05; **: *p* < 0.01; ***: *p* < 0.001.

To investigate in more details the correlations between CpG methylation and exonic evolutionary rates, we evaluated Spearman’s correlations between mCG density and each of the three evolutionary measurements (*d*_N_, *d*_S_ and the *d*_N_/*d*_S_ ratio) separately for the four sperm methylome datasets. Figure 3 shows that for first exons, mCG density has weak positive correlations with *d*_N_, *d*_S_ and the *d*_N_/*d*_S_ ratio. This observation suggests that the mutagenic effect of CpG methylation influences both synonymous and nonsynonymous sites in first exons, but the effects may be relatively small. For last exons, the Spearman’s coefficients of correlation (ρ) are consistently negative between mCG density and each of *d*_N_, *d*_S_, and the *d*_N_/*d*_S_ ratio across datasets. This observation is somewhat surprising because a negative mCG density-*d*_N_ (or mCG density-*d*_S_) correlation indicates that a higher level of CpG methylation is accompanied with a reduced rate of sequence evolution. In mammals, by comparison, the mCG density-*d*_S_ correlation is positive, while the mCG density-*d*_N_ and mCG density-*d*_N_/*d*_S_ correlations are negative in last exons [12]. Meanwhile, for internal exons, the mCG density-*d*_S_ correlations are consistently negative across datasets. However, the mCG density-*d*_N_ correlations are weakly negative in this exon group. Unexpectedly, the mCG density-*d*_N_/*d*_S_ ratio correlations are positive in internal exons. These observations appear to suggest that the synonymous sites in internal exons are subject to certain mCG-associated selective constraints, thus heavily methylated exons tend to have lower *d*_S_ values. However, such selective constraints may be weaker for the nonsynonymous sites in the same exonic regions. The decrease in *d*_S_ and the relative stasis in *d*_N_ lead to the increase in the *d*_N_/*d*_S_ ratio as mCG density increases in internal exons (Figure 3). These results also differ from what were reported for mammals, where the mCG density-*d*_S_ correlation is positive, and the mCG- *d*_N_ and mCG density-*d*_N_/*d*_S_ ratio correlations are both negative in internal exons [12]. The differences in the mCG density-evolutionary rate correlations between *A. thaliana* and mammals suggest that the biological roles of CpG methylation may have diverged between the two lineages. We also conducted the same analyses while controlling for four potential confounding factors (CpG density, G + C content, exon length, constitutive (CSE) or alternative exon (ASE) type) [12]. The results remain virtually the same (Additional file 2).

### The associations between exon length and exonic sequence evolution

**Figure 4:**
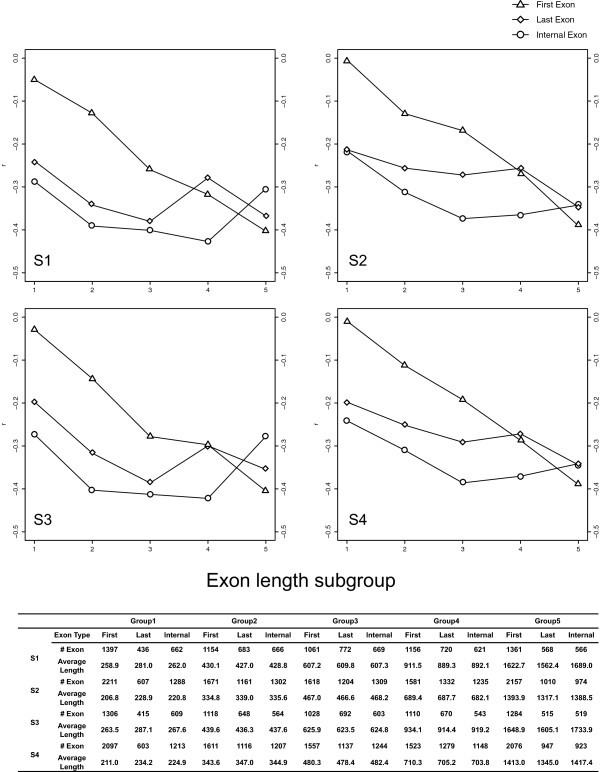
**The Pearson’s coefficient of correlations between mCG density and the CpG O/E ratio of first, last, and internal coding exons of five length subgroups (Subgroups 1 ~ 5) in the four analyzed sperm methylome datasets.** Subgroup 1 includes the shortest and subgroup 5 includes the longest exons.

We have shown that the mCG density-CpG O/E correlation is the most marked in internal exons. One important question is whether this observation has actually resulted from certain sampling biases. We noticed that our filtering criteria for dealing with the methylome data (e.g. the exon must contain > =10 sampled CpGs; see Methods) tend to retain longer exons in the datasets. The average lengths of the selected exons in S1 ~ S4 range from 615.8 to 783.5 base pairs (bp), which are considerably longer than the previously reported exome-wide average of ~250 bp in *A. thaliana*[15] and the background value (282.9 bp) in this study (Table 1). To investigate whether exon length has affected our results, we divided each of the methylome datasets (S1 ~ S4) further into five subgroups of approximately equal sizes according to exon length, and evaluated the mCG density-CpG O/E correlations separately for first, last, and internal exons for each length subgroup. Surprisingly, as shown in Figure 4, first exons show a clear length-dependent decrease in the *r* value between mCG density and the CpG O/E ratio, indicating stronger mutagenic effects of CpG methylation on longer first exons*.* By comparison, internal and last exons do not show similarly clear trends.

Since exon length is related to the mutagenic effect of CpG methylation, we then ask whether exon length is correlated with the evolutionary rates of first, last, and internal exons. Accordingly, we evaluated Spearman’s correlations between exon length and *d*_N_, *d*_S_, and the *d*_N_/*d*_S_ ratio separately for the three exon groups. Of note, here we take the background dataset (Table 1) instead of the methylome datasets to avoid potential sampling biases. Interestingly, Table 2 (“Original”) shows that for first exons, exon length is significantly correlated with both *d*_N_ and *d*_S_, although the ρ value is smaller in the exon length-*d*_N_ correlation. Meanwhile, the exon length-*d*_N_/*d*_S_ ratio correlation is statistically insignificant for this exon group. This observation seems to suggest that the increased mutagenic effect of CpG methylation (Figure 4) in longer exons has led to increases in both *d*_N_ and *d*_S_ in this exon group. This conjecture, however, is only partly true, as will be shown later. Meanwhile, for last and internal exons, the correlations between exon length and the three evolutionary measurements are all statistically significant. Yet a noteworthy difference between last and internal exons is that the ρ value of the exon length-*d*_N_ correlation is about four times larger than that of the exon length-*d*_S_ correlation for internal exons. This difference in ρ value is smaller in last exons.

**Table 2:** **The Spearman’s coefficient of correlation (ρ) between exon length and the *d***_**N**_**/*d***_**S **_**ratio, *d***_**N**_**, and *d***_**S **_**before (upper row; “Original”) and after (lower row; “Control”) controlling for four potential confounding factors (ASE/CSE exon type, proprtion of repetitive elements/disordered regions, and exonic expression level)**

		**First Exon**		**Last Exon**		**Internal Exon**	
		**ρ**	**p-value**	**ρ**	**p-value**	**ρ**	**p-value**
D_N/_D_S_	Original	−0.004	0.6126	0.090	<2.2e-16	0.156	<2.2e-16
	Control	−0.004	0.41741	0.090	1.34E-68	0.146	0
d_N_	Original	0.085	<2.2e-16	0.139	<2.2e-16	0.161	<2.2e-16
	Control	0.085	1.23E-68	0.140	9.64E-166	0.153	0
d_S_	Original	0.0187	<2.2e-16	0.112	<2.2e-16	0.039	<2.2e-16
	Control	0.0188	0	0.103	1.32-108	0.043	9.23E-67

Note that this table is based on the background dataset.

The above observations may be confounded by other biological factors. For example, ASEs are known to have increased *d*_N_ and the *d*_N_/*d*_S_ ratios as compared with CSEs [8,10,18,19]. Therefore, the increase in *d*_N_ and the *d*_N_/*d*_S_ ratio in longer exons might have resulted from an increase in the proportion of ASEs. Meanwhile, the proportion of repetitive elements (in terms of length) is also correlated with evolutionary rates because these elements are subject to relaxed selective constraints [20]. Similar comments also apply to intrinsically disordered protein regions [21-23]. The next factor to consider is exonic expression level, which has been shown to be an important determinant of *d*_N_ and the *d*_N_/*d*_S_ ratio [6,7]. We thus conducted partial Spearman’s correlation analyses while simultaneously controlling all of these four factors (the ASE/CSE exon type, proportion of repetitive elements/disordered regions, and exonic expression level). As shown in Table 2 (“Control”), the results remain virtually the same.

The last but a critical factor to control is the level of CpG methylation. To evaluate the influence of CpG methylation, we have to employ the sperm methylome datasets (S1 ~ S4), which include considerably fewer but longer exons as compared with the background dataset (Table 1). Using the four sperm methylome datasets, we again conducted partial Spearman’s correlation analysis between exon length and evolutionary rates while simultaneously controlling for mCG density, ASE/CSE exon type, proportion of repetitive elements/disordered region, and exonic expression level. As shown in Additional file 3, for first exons, the results are similar but the ρ values are decreased. This observation indicates that the length dependence of mCG-related mutagenic effect in first exons (Figure 4) accounts for part but not all of the length dependence of *d*_N_ and *d*_S_ in this exon group. Meanwhile, for last exons, the exon length-*d*_S_ correlation becomes statistically insignificant in S2 and S4, whereas the exon length-*d*_N_ and the exon length-*d*_N_/*d*_S_ correlations remain statistically significant with decreased ρ values. These results imply that mCG-related mutations may account for part of the length dependence of *d*_S_, *d*_N_, and the *d*_N_/*d*_S_ ratio in last exons. However, the decreases in ρ value and the level of statistical significance may also be ascribable to the decrease in sample size and the bias in exon length. By comparison, for internal exons, all of the correlations remain statistically significant with two notable changes as compared with the results in Table 2: (1) the ρ values of the exon length-*d*_N_ and the exon length-*d*_N_/*d*_S_ correlations are increased; and (2) the ρ values of the exon length-*d*_S_ correlations turn negative. Therefore, for internal exons, mCG-related mutations appear to be an important factor affecting *d*_S_. Nevertheless, mCG-related mutations cannot explain the length dependence of *d*_N_ and the *d*_N_/*d*_S_ ratio in this exon groups.

Taken together, our results indicate that the correlations between exon length and the evolutionary measurements (*d*_N_, *d*_S_ and the *d*_N_/*d*_S_ ratio) are unaffected by the ASE/CSE exon type, proportion of repetitive elements/disordered region, and exonic expression level in any of the three exon groups. However, the level of CpG methylation may account for part of the exon length-evolutionary rate correlations differentially for first, last, and internal exons. In summary, exon length appears to be a CpG methylation-independent indicator for *d*_N_ in all of the three exon groups, and for the *d*_N_/*d*_S_ ratio in last and internal exons of *A. thaliana*.

## Discussion

We have shown that for the coding sequences of *A. thaliana*, the mutagenic effects of CpG methylation differ between exons at different relative positions. Among the three compared exon groups (first, last, and internal), the highest CpG methylation level and the strongest mutagenic effect of CpG methylation both occur in internal coding exons (Figure 1 and Additional file 1) despite the most stringent selective constraint (lowest *d*_N_/*d*_S_ ratio) on this exon group (Figure 2). First coding exons, quite to the opposite, have the lowest level of CpG methylation and suffer the weakest mutagenic effect of CpG methylation, yet evolve the most rapidly. Interestingly, we show that mCG density is (weakly) positively correlated with *d*_S_, *d*_N_, and *d*_N_/*d*_S_ ratio in first exons, yet the same correlations are significantly negative for last exons. For internal exons, the correlations are negative, weakly negative, and positive for *d*_S_, *d*_N_, and *d*_N_/*d*_S_ ratio, respectively (Figure 4 and Additional file 2). The mutagenic effect of CpG methylation cannot fully explain these observations. Apparently, selection pressure has played a major role here. We have previously reported that in mammals, CpG methylation may have different biological roles in first, last, and internal coding exons [12]. Similar comments may also apply to *Arabidopsis* species – that first exons are more liable to the mutagenic effects, yet the other two exon groups are more affected by the regulatory functions of CpG methylation. Noticeably, however, the correlations between mCG density and evolutionary rates actually diverge significantly between *Arabidopsis* species and mammals [12]. One riveting difference is that for internal exons, the mCG density-*d*_S_ and mCG density-*d*_N_/*d*_S_ correlations are quite to the opposite between the two lineages. Such divergences appear to suggest that the biological roles of CpG methylation in coding exons have diverged significantly between the two lineages.

We also report here that exon length is an indicator of evolutionary rates of coding exons in *Arabidopsis* species. And this is not confounded by the ASE/CSE exon type, the proportion of repetitive elements, the proportion of intrinsically disordered regions, or exonic expression level. One may suspect that this observation has resulted from alignment errors, leading to increased *d*_N_ and *d*_N_/*d*_S_ ratios in longer exons. However, this is unlikely to be the case for two reasons. Firstly, the compared species - *A. thaliana* and *A. lyrata* - are very closely related. The median *d*_N_ value of first exons (which evolve the most rapidly among the three groups) is smaller than 0.03 (Figure 2). Alignment errors may be a minor issue for sequence pairs with such a high level of similarity. Secondly, the length dependence of *d*_N_ and *d*_N_/*d*_S_ ratio is unlikely to result from the alignment between paralogous exonic sequences. This is because to observe such length dependence, we should have systematically aligned orthologous sequences for shorter exons but paralogous sequences for longer exons. We perceive no possible reasons why this may happen. Another possible explanation for the length dependence of *d*_N_ and *d*_N_/*d*_S_ ratio is annotation error. However, this may not be a major problem judging from the small evolutionary rates as shown in Figure 2.

The coding exons of animal and plant genes differ from each other in a number of biological features. One example is microRNA (miRNA) targeting sites. Previous studies have reported that genes targeted by more miRNAs tend to be under stronger selective constraints [24-26]. A recent study indicated that in mammals, approximately 2% of the synonymous sites were selectively constrained for such regulatory sequences as splicing motifs, enhancers, and miRNA target sites [27]. For *A. thaliana*, it was predicted that ~75% of miRNA target sites were located in CDS [28]. In comparison, only 53.4% and 56.5% of miRNA targets were predicted to reside in CDS in human and mouse, respectively [29]. One important question is whether differential miRNA targeting is the true reason for the differences in the mCG density-evolutionary rate correlations between *Arabidopsis* species and mammals (Figure 3, [12]). Recall that the differences between the two lineages lie mainly in the mCG density-*d*_S_ correlations in internal and last exons. These correlations are significantly positive in mammals but negative in *Arabidopsis*. This divergence implies that for internal and last exons in mammals, the principal biological role of mCG is mutagenesis. In *Arabidopsis*, however, mCG density may be associated with other selection-constrained biological functions. If the divergence in mCG density-*d*_S_ correlations is to be ascribed to the higher proportion of miRNA target sites in the CDS of *Arabidopsis*, three prerequisites should be fulfilled: (1) in the internal and last exons of *Arabidopsis*, mCG density must be positively correlated with the probability of miRNA targeting; (2) miRNA targeting must be significantly constrained by selection in the two exon groups of *Arabidopsis*; and (3) this miRNA targeting-related selection affects only synonymous sites in internal and last exons of *Arabidopsis*. An example of miRNA-mediated DNA methylation has been reported for rice [30]. The authors discovered that a specific group of 24-nucleotide (nt) miRNAs could mediate DNA methylation within a ~80-nt region around the target sites. However, only five such targets were identified. And most of the methylation occurred in the CHH or CHG context [30]. A follow-up study published lately showed that 65 of 24-nt miRNAs exhibited elevated CHH methylation (but not CpG methylation) around their target sites [31]. These studies imply that miRNA targeting may lead to an increased level of DNA methylation in the gene body of plants (which, in fact, was also observed in human [32]). Of note, nevertheless, each miRNA was predicted to have only one target site in the target gene. Furthermore, only 13 of the 65 target sites were located in CDS [31]. Meanwhile, a recent study suggested that the miRNA target sites in CDS were subject to negative selection [33]. These observations seem to suggest a connection between miRNA targeting and the mCG density-*d*_S_ correlations in plants. However, we speculate that the influences of miRNA targeting might be insubstantial for three reasons. First, only a relatively small number (tens) of miRNAs have been reported to cause DNA methylation at the target sites. And most of them occur outside of CDS. miRNA-mediated methylation in CDS thus may be uncommon. Second, the sequences that are subject to miRNA-mediated methylation account for a minority (~80 nt [30] or ~200 nt [31]) in light of the average CDS length of ~1300 bp in the *A. thaliana* genome [15]. Certainly, we cannot exclude the possibility that a methylation-inducing miRNA has multiple target sites in one gene, or that a gene is targeted by multiple methylation-inducing miRNAs. In such cases, the effects of miRNA targeting will undoubtedly be non-negligible. Nevertheless, these scenarios were not observed in the recent studies [30,31]. The overall influences of miRNA targeting on CDS methylation thus might be immaterial. Third, the identified miRNA-mediated DNA methylation occurred mostly in the CHH or CHG contexts [30,31]. Since we focus on methylation at CpG dinucleotides, the influences of miRNA-mediated methylation on our analysis should be fairly limited.

Another potential confounding factor in the mCG density-evolutionary rate analysis is the level of protein phosphorylation. Phosphorylated amino acid residues have been known to evolve more slowly than those unphosphorylated [34-37]. Since the motifs for phosphorylation differ between *Arabidopsis* and mammals [38,39], the evolutionary rates of coding exons in the two lineages may be differentially affected by phosphorylation-related constraints. However, phosphorylation occurs at amino acid residues. The selective constraints at the amino acid level influence *d*_N_ but not *d*_S_. Note that the mCG density-*d*_N_ correlations are generally similar between mammals and *Arabidopsis* (Figure 3, [12]). Therefore, phosphorylation appears to have no significant effects on the differences in the mCG density-*d*_N_ correlations between the two lineages.

One may suspect that the correlations between exon length and *d*_N_ and *d*_N_/*d*_S_ ratio have resulted from functional biases. This is because exons of different lengths may belong to genes of different functional categories. To examine this possibility, we divided the background dataset (Table 1) into five length subgroups and conducted an all-to-all pairwise comparison of gene ontology functional categories between the five subgroups of internal exons using FatiGO [40]. As shown in Additional file 4, although the five length subgroups of internal exons differ from one another in view of gene ontology annotations, we do not observe any particular trend that may cause the length dependence of *d*_N_ and *d*_N_/*d*_S_ ratio. We also examined whether the correlations between mCG density and evolutionary rate could differ between different functional categories. We classified the analyzed genes according to the third level of “Molecular Function” of Gene Ontology, and calculated the correlations for nine functional groups that included ≥ 1000 genes. Note that one gene can be assigned to multiple functional groups. The sum of genes in all of the functional groups thus outnumbers the analyzed genes. The mCG density-evolutionary rate correlations in individual functional groups are similar to what we observed in Figure 3 (Additional file 5). Therefore, functional bias may not be a major concern in our analysis.

The correlations between exon length and evolutionary rates in *Arabidopsis* species have been previously observed [7]. However, the underlying mechanism remains unclear. Here we show that first, last, and internal coding exons diverge from each other in terms of the exon length-*d*_N_/*d*_S_ ratio correlation – the correlation is stronger in internal exons than in last exons, and is statistically insignificant in first exons. The length dependence of *d*_N_/*d*_S_ ratio in last and internal exons remains statistically significant after controlling for potential confounding factors (the ASE/CSE exon type, the content of repetitive elements/disordered region, exonic expression level, and the level of CpG methylation). Of note, for last and internal exons, this length dependence occurs because longer exons have a larger increase in *d*_N_ than in *d*_S_ when compared with shorter exons. This increase in *d*_N_ is probably unrelated to structural-functional reasons, for the proportion of disordered protein region (which is an indicator of protein structural flexibility and is strongly associated with the content of protein domains) does not significantly affect the exon length-*d*_N_/*d*_S_ ratio correlations. It will be interesting to test the evolutionary neutrality of exons of different lengths when adequate polymorphism data become available.

Meanwhile, it has been recently reported that in human, transcription factor binding sites (TFBS) frequently reside in coding exons, and may significantly affect the evolution of these exonic sequences [41]. The same comment may also apply to *A. thaliana*. However, currently no base-resolution TFBS datasets are available for *A. thaliana*. We may revisit this issue and investigate whether the density of TFBS is associated with the observed length dependence of *d*_N_ and *d*_N_/*d*_S_ ratio when such datasets are accessible.

One important issue is that we analyzed only one plant species in this study. Whether the observations in *A. thaliana* can be applied to other plant species remains unknown. To address this issue, we retrieved three genome-scale methylome datasets of rice (*Oryza sativa* L. ssp. *japonica*). Two of the datasets were derived from young panicles [42], and the other was derived from leaves [43] (Additional file 6). Our analysis confirmed the mutagenic effect of mCG on coding exons and the stronger mutagenic effect on non-first exons than on first exons in rice (Additional file 7). The evolutionary rates of first, last, and internal exons were similar to what we observed in *A. thaliana* (Additional file 8). Intriguingly, however, the correlations between mCG density and evolutionary rates were fairly different between rice (Additional file 9) and *A. thaliana* (Figure 3). Particularly, in view of the mCG density-*d*_S_ correlations in last and internal exons, rice was similar to mammals [12] but not to *A. thaliana*. Of note, the rice methylome data were derived from panicles and leaves but not gamete cells. Whether the identified mCGs and the associated substitutions are heritable is therefore questionable. To be sure, we cannot rule out the possibility that the differences in mCG density-*d*_S_ correlations between *A. thaliana* and rice represent genuine divergences in the biological roles of mCG. Adding to the complexity of this issue is that the domesticated rice (*O. japonica*) has been artificially selected. It will be interesting to re-examine this topic when the gamete methylome datasets of both cultivated and wild rice are available.

## Conclusions

The mammal-*Arabidopsis* divergence in the association between DNA methylation and coding exon evolution is unexpected. DNA methylation is a major source of genomic sequence mutation on one hand, and an important transcriptional/splicing regulator on the other hand. Our results imply that this balance between biological roles of DNA methylation in coding exons may have differed significant between *Arabidopsis* and mammals in a length- and position-dependent manner. The detailed evolutionary mechanisms and functional outcomes are worth further explorations.

## Methods

### Measurement of CpG methylation level and the CpG O/E

The genome-scale, single base-resolution DNA methylation datasets of *A. thaliana* sperm were retrieved from a recent study [44] under accession number SRX156133 (Table 1). The bisulfite sequence reads were mapped to the genome of *A. thaliana* (TAIR10), and the methylated CpGs being identified by BS-Seeker [45] with default parameters. To ensure data quality, only the CpG dinucleotides that are covered by ≥5 bisulfite reads were retained (such CpG dinucleotides are designated as “sampled CpGs”). The methylation status of a CpG was represented as the percentage of reads that support the methylation of this CpG site. Only the CpGs with a methylation frequency of ≥80% were regarded as methylated [46,47], and designated as “mCGs”. Since the accuracy of evolutionary rate estimates may be compromised in the case of short exons (e.g., <50 bp) [18,21,48], we only considered the CDSs that are longer than 80 bp and contain ≥10 sampled CpGs to ensure that the CDSs contain sufficient information. Here we focus on CpG methylation because the other types (CHG and CHH) of methylation are relatively rare [17], and may have only minor effects on the evolution of *Arabidopsis* exons.

The level of CpG methylation of a particular exonic region was represented by the “mCG density”, which was measured by calculating the number of mCGs per 100 CpG dinucleotides, and was defined as $\;\mathrm{mCG}\;\mathrm{density}=\frac{\mathrm{number}\;\mathrm{of}\;\mathrm{mCGs}\times 100}{\mathrm{number}\;\mathrm{of}\;\mathrm{all}\;\mathrm{CpGs}\;\mathrm{sampled}}$.

The CpG O/E was defined as $\mathrm{CpG}\;O/E=\frac{P_{\mathrm{CpG}}}{P_{c}\times P_{G}}=\frac{\mathrm{number}\;\mathrm{of}\;\mathrm{observed}\;\mathrm{CpG}\times \mathrm{exon}\;\mathrm{length}}{\mathrm{number}\;\mathrm{of}\;C\times \mathrm{number}\;\mathrm{of}\;G}$, where *P*_*CpG*_, *P*_*C*_, and *P*_*G*_ represent the frequency of CpG dinucleotides, C nucleotides, and G nucleotides, respectively.

### Classification of coding exons

The *A. thaliana* gene annotations and the corresponding coding sequences were downloaded from the Ensembl genome browser at http://www.ensembl.org/. The CDSs that overlap with non-coding RNAs or pseudogenes were excluded. Single-exon genes were also excluded. According to the relative positions of exons in the Ensembl-annotated genes, the retrieved coding exonic regions were divided into three groups: first, internal, and last exons. Briefly, all of the transcript isoforms of a gene were collated (except for those that overlapped non-coding RNAs or pseudogenes), and the coordinates of the exons were compared. The coding exon that was closest to the most downstream 5’UTR and the most upstream 3’UTR was classified as the first and last coding exon, respectively. However, in the case where a stand-alone 5’UTR exon was followed by a second 5’UTR juxtaposed to a coding exon, this coding exon was excluded. This is because in this case, the first coding exon is not part of the most upstream exonic region. The same comment also applied to the last exon. The remaining exons that were neither first nor last coding exons were considered as internal exons. The retrieved exons were also classified into constitutively and alternatively spliced exons (CSEs and ASEs, respectively) according to whether they were always present in different transcript isoforms of a gene.

### Measurement of exonic expression level

The transcriptome data for *A. thaliana* pollen derived from a recent study [49] were retrieved from the Gene Expression Omnibus database under accession number SRP022162. The sequencing reads were mapped to the *A. thaliana* genome by using TopHat 2 [50], and then analyzed by using eXpress [51] to obtain exonic expression levels.

### Predictions of intrinsically disordered regions and repetitive elements

The genomic and peptide sequences of *A. thaliana* retrieved from the ENSEMBL Plants website were submitted to RepeatMasker [52] and Disopred [53], respectively, for predictions of repetitive elements and intrinsically disordered regions. The prediction tools were applied with default parameters. The proportions of exonic regions that overlapped repetitive elements and disordered regions were then calculated separately.

### Calculation of evolutionary rates

The one-to-one gene orthology between *A. thaliana* and *A. lyrata* was retrieved from ENSEMBL Plants (Version 18). The protein sequences of the orthologous genes were aligned using MUSCLE [54] and then back-translated to nucleotide sequences. The aligned sequences were then separated exon-wise according to the annotations of ENSEMBL. The exonic sequence alignments were checked for the correctness of reading frame before being submitted to the CodeML program of PAML4 [55] for the calculations of *d*_N_, *d*_S_ and the *d*_N_/*d*_S_ ratio.

## Abbreviations

ASE: Alternative exon; CDS: Coding sequence; CpG methylation: The level of DNA methylation at CpG dinucleotides; CpG O/E ratio: The observed-to-expected ratio of the number of CpG dinucleotides; CSE: Constitutive exon; *d*_N_: Nonsynonymous substitution rate; *d*_S_: Synonymous substitution rate; mCG: Methylated CpG dinucleotide; TFBS: Transcription factor binding site; UTR: Untranslated region.

## Competing interests

The authors declare that they have no competing interests.

## Authors’ contributions

Conceived the study: FCC; designed the research: FCC; conducted data collection and analysis: HYL and MKH; data interpretation: FCC and TJC; wrote the manuscript: FCC and TJC. All authors read and approved the final manuscript.

## Supplementary Material

Additional file 1 **The median mCG densities of first, last, and internal coding exons in different methylation datasets.** All pairwise differences between exon groups in each dataset are statistically significant (*p* < 0.001, by Wilcoxon Rank Sum Test). Click here for file

Additional file 2 **The Spearman’s coefficients of correlation between mCG density and the *d***_**N**_**/*d***_**S **_**ratio, *d***_**N**_**, and *d***_**S **_**based on S1 ~ S4 datasets after controlling for four potential confounding factors (CpG density, G + C content, exon length, ASE/CSE exon type).** *: *p* < 0.05; **: *p* < 0.01; ***: *p* < 0.001. (DOC 52 kb) Click here for file

Additional file 3 **The Spearman’s coefficients of correlation and *p *** **values between exon length and *d***_**N**_**/*d***_**S**_** ratio, *d***_**N**_**, and *d***_**S **_**for first, last, and internal exons before (“Original”) and after (“Control”) controlling for five potential confounding factors (the ASE/CSE exon type, the proportion of repetitive elements/disordered regions, exonic expression level, and mCG density).** Click here for file

Additional file 4 **Pairwise comparison between length subgroups of internal coding exons in view of Gene Ontology functional categories.** Note that here the “function” of an exon is the function of the gene it resides. The Y axis indicates the percentage of each length subgroup in a specific functional category. The table at the bottom shows whether the differences between subgroups are statistically significant. Lighter grey shading indicates that the former subgroup is relatively enriched. Darker grey shading indicates the contrary. ***: *p* < 0.001. The numbers on top of the table indicate the percentages of genes in a specific functional category over all of the analyzed genes. Click here for file

Additional file 5 **The Spearman’s coefficients of correlation between mCG density and the *d***_**N**_**/*d***_**S **_**ratio, *d***_**N**_**, and *d***_**S **_**for different Gene Ontology functional categories (Level 3 of Molecular Function).** *: *p* < 0.05; **: *p* < 0.01; ***: *p* < 0.001. Click here for file

Additional file 6 The methylome datasets of rice (panicles: P1 and P2; leaves: L). Click here for file

Additional file 7 **Pearson’s coefficients of correlation between mCG density and the CpG O/E ratio in (A) different methylome datasets (panicles: P1and P2; leaves: L); (B) first, last, and internal coding exons in different methylome datasets of rice.** ***: *p* < 0.001. Click here for file

Additional file 8 **The evolutionary rates (*d***_**N**_**/*d***_**S **_**ratio, *d***_**N**_**, and *d***_**S**_**) of first, last, and internal coding exons in different methylome datasets of rice (panicles: P1and P2; leaves: L).** The curves with stars indicate statistically significant difference. **: *p* < 0.01; ***: *p* < 0.001, by Wilcoxon Rank Sum Test. Click here for file

Additional file 9 **The Spearman’s coefficients of correlation between mCG density and the *d***_**N**_**/*d***_**S **_**ratio, *d***_**N**_**, and *d***_**S **_**based on different methylome datasets of rice (panicles: P1and P2; leaves: L).** *: *p* < 0.05; **: *p* < 0.01; ***: *p* < 0.001. Click here for file
