# Supplementary material for: The evolution of the coding exome of the Arabidopsis species - the influences of DNA methylation, relative exon position, and exon length
Source: BMC Evol Biol. 2014 Jun 25;14:145. doi: 10.1186/1471-2148-14-145 (PMC4079183; doi:10.1186/1471-2148-14-145)
Supplement: Supplementary file 3 [file 1471-2148-14-145-S3.doc]

			First Exon		Last Exon		Internal Exon	
			ρ	p-value	ρ	p-value	ρ	p-value
S1	d_N/d_S	Original	-0.007	5.99E-01	0.062	5.07E-04	0.228	< 2.2e-16
		Control	-0.007	2.43E-01	0.063	2.09E-15	0.228	2.71E-200
	d_N	Original	0.039	2.34E-03	0.068	1.33E-04	0.183	< 2.2e-16
		Control	0.038	1.70E-11	0.069	2.30E-18	0.183	2.57E-128
	d_S	Original	0.099	8.33E-15	-0.016	3.58E-01	-0.151	< 2.2e-16
		Control	0.098	2.10E-66	-0.016	4.68E-02	-0.150	4.86E-85
S2	d_N/d_S	Original	-0.007	4.81E-01	0.066	1.47E-06	0.212	< 2.2e-16
		Control	-0.007	1.11E-01	0.067	4.98E-28	0.212	0
	d_N	Original	0.053	4.07E-07	0.079	7.07E-09	0.193	< 2.2e-16
		Control	0.052	3.08E-29	0.081	1.07E-39	0.193	2.80E-283
	d_S	Original	0.126	< 2.2e-16	0.003	8.43E-01	-0.088	4.72E-13
		Control	0.125	3.59E-162	0.003	6.32E-01	-0.087	1.31E-57
S3	d_N/d_S	Original	-0.013	3.34E-01	0.064	5.13E-04	0.237	< 2.2e-16
		Control	-0.013	2.94E-02	0.065	1.84E-15	0.237	4.98E-194
	d_N	Original	0.029	2.57E-02	0.065	4.09E-04	0.193	< 2.2e-16
		Control	0.029	9.02E-07	0.067	4.94E-16	0.194	4.98E-128
	d_S	Original	0.094	4.54E-13	-0.026	1.61E-01	-0.143	5.75E-15
		Control	0.094	4.24E-58	-0.025	2.07E-03	-0.142	6.65E-68
S4	d_N/d_S	Original	-0.007	5.40E-01	0.057	5.07E-05	0.214	< 2.2e-16
		Control	-0.007	1.61E-01	0.058	1.86E-20	0.214	0
	d_N	Original	0.051	1.52E-06	0.071	3.88E-07	0.192	< 2.2e-16
		Control	0.050	1.91E-26	0.072	4.71E-31	0.193	1.93E-264
	d_S	Original	0.124	< 2.2e-16	0.008	5.46E-01	-0.091	4.69E-13
		Control	0.123	1.19E-149	0.009	1.63E-01	-0.090	1.11E-57

Additional File 3
