# Supplementary material for: The evolution of the coding exome of the Arabidopsis species - the influences of DNA methylation, relative exon position, and exon length
Source: BMC Evol Biol. 2014 Jun 25;14:145. doi: 10.1186/1471-2148-14-145 (PMC4079183; doi:10.1186/1471-2148-14-145)
Supplement: Supplementary file 4 [file 1471-2148-14-145-S4.doc]

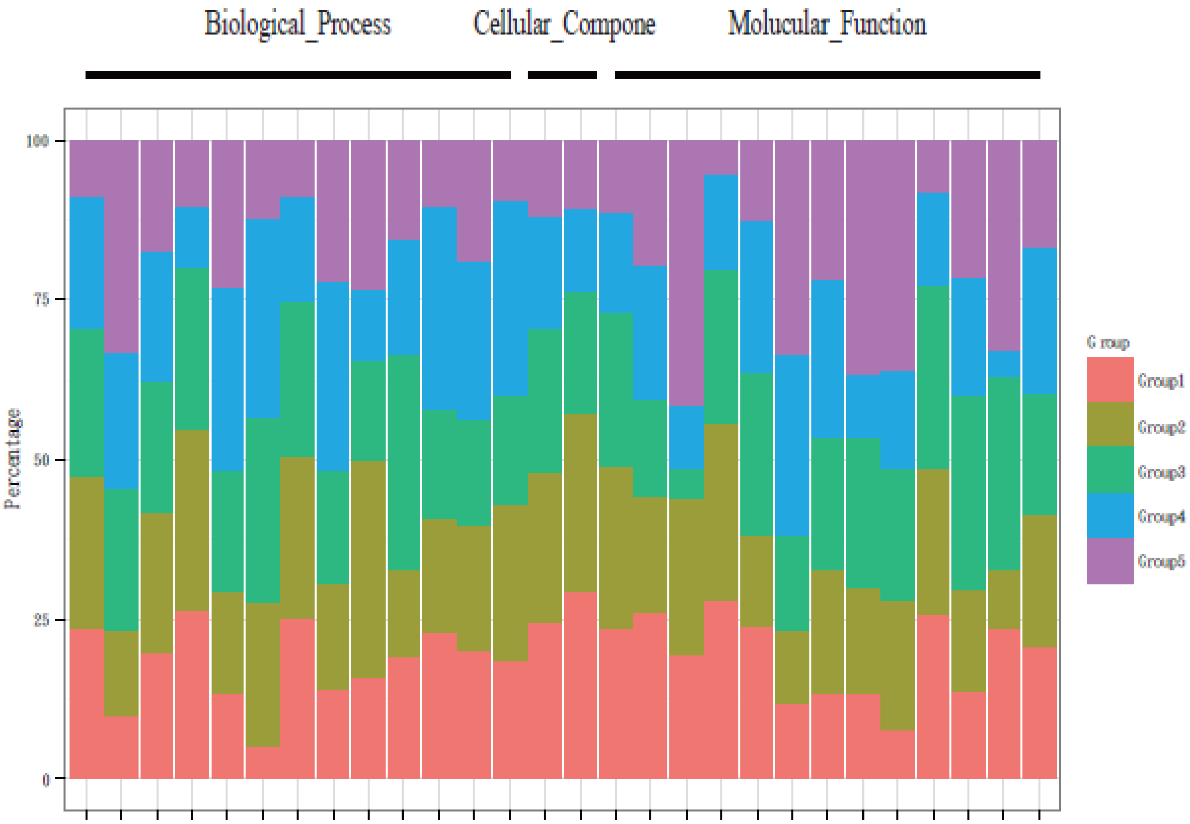

	1	2	3	4	5	6	7	8	9	10	11	12	13	14	15	16	17	18	19	20	21	22	23	24	25	26	27	28
	alcohol metabolic process	cell adhesion	cell communication	cell cycle	cell death	cell recognition	cellular localization	immune response	multicellular organismal development	oxidation reduction	response to biotic stimulus	response to external stimulus	secondary metabolic process	protein complex	vesicle	cofactor binding	drug transporter activity	enzyme inhibitor activity	isomerase activity	oxidoreductase activity	oxygen binding	signal transducer activity	structural constituent of cell wall	structural constituent of cytoskeleton	structural constituent of ribosome	tetrapyrrole binding	transcription activator activity	transmembrane transporter activity
(%)	3.7	0.4	10.4	3.5	0.9	0.1	6.1	1.3	11.1	4.9	2.3	1.9	2.1	15.4	1.1	4.9	1.1	0.2	2.6	9.4	0.3	4.5	0.1	0.1	1.2	0.6	0.7	9.2
1 vs. 2																												
1 vs. 3			***														***					***				***		
1 vs. 4					***	***	***	***			***		***	***	***				***		***	***			***			
1 vs. 5	***		***	***	***	***	***	***				***		***	***	***		***	***	***	***	***			***	***	***	***
2 vs. 3																										***		
2 vs. 4					***		***	***				***	***	***	***				***		***	***						***
2 vs. 5	***		***	***	***		***	***	***			***				***	***	***	***		***	***	***	***	***	***		***
3 vs. 4					***		***	***						***					***		***	***			***			***
3 vs. 5	***	***	***	***	***		***	***		***						***	***		***	***	***	***		***	***		***	***
4 vs. 5	***	***	***																***	***		***						

Additional File 4.
