# Supplementary material for: The evolution of the coding exome of the Arabidopsis species - the influences of DNA methylation, relative exon position, and exon length
Source: BMC Evol Biol. 2014 Jun 25;14:145. doi: 10.1186/1471-2148-14-145 (PMC4079183; doi:10.1186/1471-2148-14-145)
Supplement: Supplementary file 6 [file 1471-2148-14-145-S6.doc]

Additional File 6. The rice methylome datasets.

Dataset	# Gene	# Exon	Read Depth	Ave. mCG density (per 100 Sampled CpG)	Ave.CpG density (per 100 bp)	# First/Last/Internal	Ave. length
P1	11192	18959	25	11.42	10.33	7129/4968/6862	521.71
P2	9571	14737	13	13.43	10.54	5358/4255/5124	581.20
L	8871	14748	75	19.04	7.19	3740/3490/7518	544.12
