# Supplementary material for: The evolution of the coding exome of the Arabidopsis species - the influences of DNA methylation, relative exon position, and exon length
Source: BMC Evol Biol. 2014 Jun 25;14:145. doi: 10.1186/1471-2148-14-145 (PMC4079183; doi:10.1186/1471-2148-14-145)
Supplement: Supplementary file 9 [file 1471-2148-14-145-S9.doc]

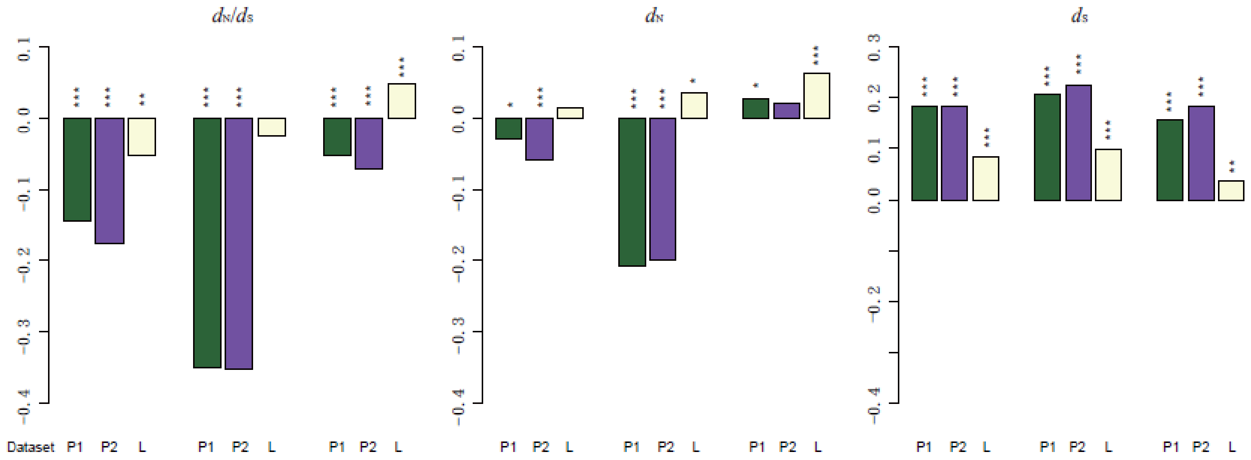

Additional File 9
